# Paediatric diabetes subtypes in a consanguineous population: a single-centre cohort study from Kurdistan, Iraq

**DOI:** 10.1007/s00125-023-06030-2

**Published:** 2023-10-28

**Authors:** Shenali A. Amaratunga, Tara Hussein Tayeb, Rozhan N. Muhamad Sediq, Fareda K. Hama Salih, Petra Dusatkova, Matthew N. Wakeling, Elisa De Franco, Stepanka Pruhova, Jan Lebl

**Affiliations:** 1 Department of Paediatrics, 2nd Faculty of Medicine, Charles University in Prague and Motol University Hospital, Prague, Czech Republic; 2 Diabetic Clinic, Dr Jamah Ahmad Rashed Hospital, Sulaimani, Kurdistan Iraq; 3 Department of Paediatrics, College of Medicine, Sulaimani University, Sulaimani, Kurdistan Iraq; 4 Clinical and Biomedical Sciences, University of Exeter Faculty of Health and Life Sciences, Exeter, UK

**Keywords:** Consanguineous population, Consanguinity, Diabetes genes, Genetics, Monogenic diabetes, Neonatal diabetes, Paediatric diabetes, Syndromic diabetes

## Abstract

**Aims/hypothesis:**

Monogenic diabetes is estimated to account for 1–6% of paediatric diabetes cases in primarily non-consanguineous populations, while the incidence and genetic spectrum in consanguineous regions are insufficiently defined. In this single-centre study we aimed to evaluate diabetes subtypes, obtain the consanguinity rate and study the genetic background of individuals with syndromic and neonatal diabetes in a population with a high rate of consanguinity.

**Methods:**

Data collection was carried out cross-sectionally in November 2021 at the paediatric diabetic clinic, Dr Jamal Ahmad Rashed Hospital, in Sulaimani, Kurdistan, Iraq. At the time of data collection, 754 individuals with diabetes (381 boys) aged up to 16 years were registered. Relevant participant data was obtained from patient files. Consanguinity status was known in 735 (97.5%) participants. Furthermore, 12 families of children with neonatal diabetes and seven families of children with syndromic diabetes consented to genetic testing by next-generation sequencing. Prioritised variants were evaluated using the American College of Medical Genetics and Genomics guidelines and confirmed by Sanger sequencing.

**Results:**

A total of 269 of 735 participants (36.5%) with known consanguinity status were offspring of consanguineous families. An overwhelming majority of participants (714/754, 94.7%) had clinically defined type 1 diabetes (35% of them were born to consanguineous parents), whereas only eight (1.1%) had type 2 diabetes (38% consanguineous). Fourteen (1.9%) had neonatal diabetes (50% consanguineous), seven (0.9%) had syndromic diabetes (100% consanguineous) and 11 (1.5%) had clinically defined MODY (18% consanguineous). We found that consanguinity was significantly associated with syndromic diabetes (*p*=0.0023) but not with any other diabetes subtype. The genetic cause was elucidated in ten of 12 participants with neonatal diabetes who consented to genetic testing (homozygous variants in *GLIS3* [sibling pair], *PTF1A* and *ZNF808* and heterozygous variants in *ABCC8* and *INS*) and four of seven participants with syndromic diabetes (homozygous variants in *INSR*, *SLC29A3* and *WFS1* [sibling pair]). In addition, a participant referred as syndromic diabetes was diagnosed with mucolipidosis gamma and probably has type 2 diabetes.

**Conclusions/interpretation:**

This unique single-centre study confirms that, even in a highly consanguineous population, clinically defined type 1 diabetes is the prevailing paediatric diabetes subtype. Furthermore, a pathogenic cause of monogenic diabetes was identified in 83% of tested participants with neonatal diabetes and 57% of participants with syndromic diabetes, with most variants being homozygous. Causative genes in our consanguineous participants were markedly different from genes reported from non-consanguineous populations and also from those reported in other consanguineous populations. To correctly diagnose syndromic diabetes in consanguineous populations, it may be necessary to re-evaluate diagnostic criteria and include additional phenotypic features such as short stature and hepatosplenomegaly.

**Graphical Abstract:**

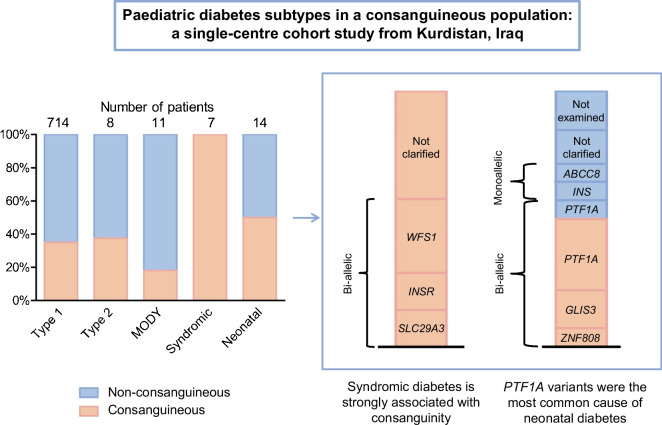


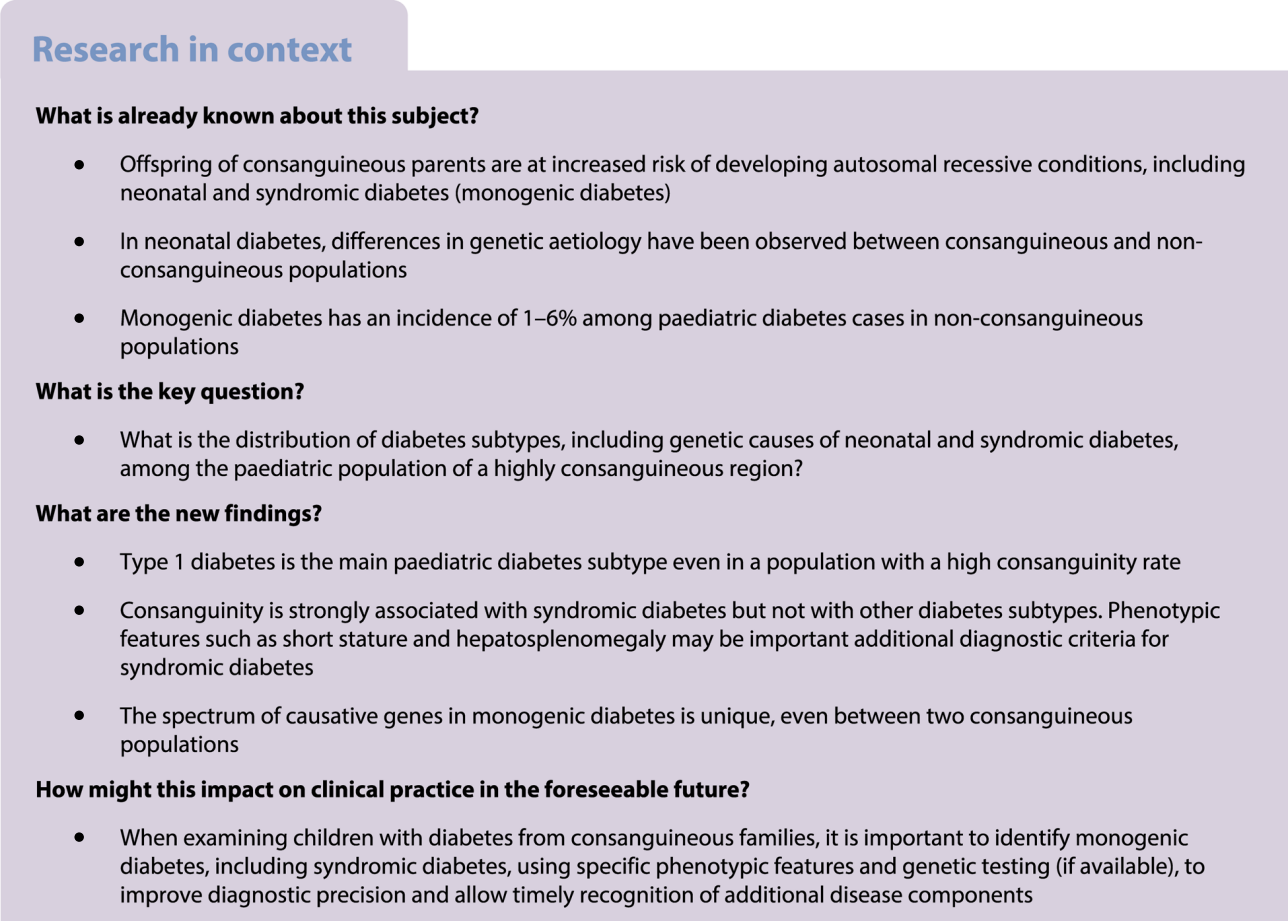


## Introduction

The majority of diabetes cases diagnosed in childhood and adolescence are of polygenic aetiology, with significant contributing environmental factors. This is the case for both type 1 diabetes, which is the predominant form, and type 2 diabetes, which occurs much less frequently but is now on the rise in some parts of the world due to an increase in the prevalence of childhood obesity [1]. Monogenic diabetes is more rare, with varied aetiology and multiple clinical forms [2].

Paediatric forms of monogenic diabetes can be divided into four distinct but partially overlapping types: MODY, which exhibits autosomal dominant transmission; neonatal diabetes, with clinical onset within the first 6 months of age; syndromic diabetes, characterised by additional non-diabetic (usually extra-pancreatic) phenotypic features; and monogenic autoimmune diabetes, which is associated with additional immune-mediated conditions.

Neonatal diabetes is clearly defined by the age at clinical onset, whereas other subtypes of monogenic diabetes may remain underdiagnosed or misdiagnosed, especially in resource-limited countries where testing of pancreatic autoantibodies is rarely carried out and cases of monogenic diabetes may be classified as type 1 or type 2 diabetes. Thus, with the exception of neonatal diabetes, the incidence of all subtypes of monogenic diabetes may be underestimated [3]. The fact that some genetic variants are phenotypically variable, with milder forms of diabetes appearing only later in adolescence or in adulthood, complicates the correct disease classification even further. Some syndromic forms of monogenic diabetes may present initially as insulin-dependent diabetes mellitus, while further features may be delayed for years, as is the case with Wolfram syndrome, or are inconsistent, such as in renal cyst and diabetes syndrome [4].

Monogenic diabetes has been estimated to account for 1–6% of all diabetes cases in European countries, which are primarily non-consanguineous [5–7]. An incidence of 6% has been reported from a large centre in Turkey but the incidence in other countries where consanguinity is prevalent is not known [8, 9]. However, the genetic aetiology of monogenic diabetes, specifically neonatal diabetes, differs significantly between areas with low and areas with high rates of consanguinity [10].

Our study centre is the sole clinic responsible for paediatric diabetes care in the entire region of Sulaimani in Kurdistan, Iraq. According to unpublished 2021 statistics available from the regional authorities in Sulaimani, the population of this region was 2.33 million, with 256,000 being children under 5 years of age. There were 46,000 live births in the region in 2021 and, according to United Nations Children’s Fund (UNICEF) data, the infant mortality rate in Iraq is 21 per 1000 live births [11]. The consanguinity rate in this region has not been documented to the authors’ knowledge, but in geographically and/or ethnically related regions it has been reported as 39–44% [12–14].

Investigations such as basic biochemical and HbA_1c_ testing are available at the clinic, but antibody assessment is carried out only in private laboratories, at the individual’s expense, and thus is available for only a few people. Furthermore, genetic testing is almost inaccessible. Taking all of these factors into account, it is very likely that monogenic diabetes in this region is underdiagnosed.

In this single-centre study, we aimed to evaluate the prevalence of clinically defined subtypes of paediatric diabetes, calculate the consanguinity rate and study the genetic background of individuals with neonatal and syndromic diabetes.

## Methods

### Data collection

A total of 754 individuals were registered at the diabetic clinic at Dr Jamal Ahmad Rashed Hospital in the region of Sulaimani in Kurdistan, Iraq, in November 2021. This is the only clinic serving the region and therefore the study sample (which is derived from the paediatric population comprising mostly Kurdish people and some Arabic individuals [self reported]) is representative of the entire population of Kurdistan, Iraq. Data were collected from these individuals with their consent, with the use of information from hand-written medical records, in the form of a cross-sectional survey. Additional data were obtained during participant check-ups and direct telephone interviews with participants’ families were carried out regarding consanguinity if this information was not available. The date of diagnosis, age at diagnosis, sex (determined during examination), type of clinically determined diabetes, insulin dosage, syndromic features (if any), anthropometric measurements, family history of diabetes and consanguinity status were recorded. Positive consanguinity was defined as children born to first, second or third cousin parents, as reported by the respective families.

The clinical determination of diabetes subtype was carried out as follows:

1. Type 1 diabetes: insulin-dependent diabetes diagnosed at age >6 months with or without diabetic ketoacidosis at disease onset, without vertical transmission of diabetes in the family, dysmorphic features or concomitant conditions, except for coeliac disease and/or autoimmune thyroid disease.
2. Type 2 diabetes: non-insulin-dependent diabetes diagnosed in children and adolescents with a BMI higher than that of peers of the same age with diabetes; features of insulin resistance; normal or high C-peptide levels; and testing negative for autoantibodies (when available).
3. Neonatal diabetes: diabetes diagnosed up to 6 months of age.
4. Syndromic diabetes: diabetes diagnosed after 6 months of age, testing negative for autoantibodies (when available) and accompanied by other significant features (dysmorphic phenotypic signs, short stature, hepato- and/or splenomegaly, known renal cysts or additional immunopathological conditions, excluding autoimmune thyroid disease and/or coeliac disease).
5. MODY: apparent vertical transmission of diabetes in families, with diabetes diagnosed at age >6 months with no diabetic ketoacidosis at disease onset.

From the information collected, association of diabetes subtypes and consanguinity status was analysed using Fisher’s two-sided exact test, with *p*<0.05 considered statistically significant.

Participants with neonatal diabetes or syndromic diabetes were offered genetic testing. Nineteen children from 17 families (12 with neonatal diabetes and seven with syndromic diabetes, including two sibling pairs) were sent for genetic testing with written parental consent. Peripheral blood for DNA extraction was collected from the children, both parents and, if possible, additional family members to allow for more extensive segregation analysis. Genetic analysis by next-generation sequencing (NGS) was carried out at the Laboratory of Molecular Genetics, Department of Paediatrics, Motol University Hospital and Charles University in Prague, Czech Republic.

### Genetic examination

Genomic DNA was extracted from peripheral blood using the QIAmp DNA Blood Mini system (Qiagen, Hilden, Germany). DNA was analysed using whole-exome sequencing (WES). WES was performed using the SureSelect Human All Exon V6+UTR kit (Agilent Technologies, Santa Clara, CA, USA) and the indexed products were sequenced by synthesis in an Illumina NextSeq 500 analyser (San Diego, CA, USA). Our bioinformatic pipeline includes evaluation of the most prevalent variants in mitochondrial DNA, as described previously [15]. Copy number variants subanalysis from raw WES data was carried out using the DECoN program [16]. Variant frequency was assessed using the Genome Aggregation Database (gnomAD) (total allele frequency across all populations) [17]. Prioritised variants were then further evaluated using the American College of Medical Genetics and Genomics (ACMG) standards and guidelines [18]. All of the variants with potential clinical significance were confirmed using Sanger sequencing, as described previously [19]. To evaluate the segregation of genetic variants within families, Sanger sequencing was performed in both parents and healthy/affected siblings (when available), with written informed consent.

Probands with neonatal diabetes without a detected causal variant using WES underwent Sanger sequencing of the *PTF1A* enhancer region and methylation-specific multiplex ligation probe-dependent amplification in order to detect the aberrant methylation and/or gene dosage of chromosomal regions 6q22, 6q24 and 11p15 linked to transient neonatal diabetes (SALSA MS-MLPA Probemix ME033-A1 TNDM, MRC Holland, Amsterdam, the Netherlands). Furthermore, WES data from probands without a detected causal variant after the previous steps were further examined using the pipeline developed at the department of Clinical and Biomedical Sciences, University of Exeter Medical School, Exeter, UK. This pipeline has been previously described and is primarily used to search for variants in candidate genes [20].

Genome-wide runs of homozygosity (ROH) were analysed in all probands from FASTQ data using SavvyCNV to verify reported consanguinity. The discovered regions elude to the per cent of the autosome covered by homozygous regions at least 3 Mbp in size [21].

### Ethics statement

Participants’ parents gave informed consent for the genetic testing reported in this paper and for the publication of related data and full-face images of participants. This study was approved by the Ethics Committee of Motol University Hospital and 2nd Faculty of Medicine, Charles University, Prague (approval no. EK-1263.1.1/19). The research was conducted ethically in accordance with the World Medical Association’s Declaration of Helsinki.

## Results

### General participant data

Data were obtained from a total of 754 children from 735 families (381 boys, 50.5%) of predominantly Kurdish origin. The consanguinity status was known for 735 participants; the remaining participants could not be contacted for further information. A total of 269 participants (36.5%) from 258 families were offspring of consanguineous families. Multiple children with diabetes from the same family all had the same diabetes subtype. Consanguinity was significantly associated with syndromic diabetes (*p*=0.0023) but not with other diabetes subtypes.

### Type 1 diabetes, type 2 diabetes and MODY

**Fig. 1:**
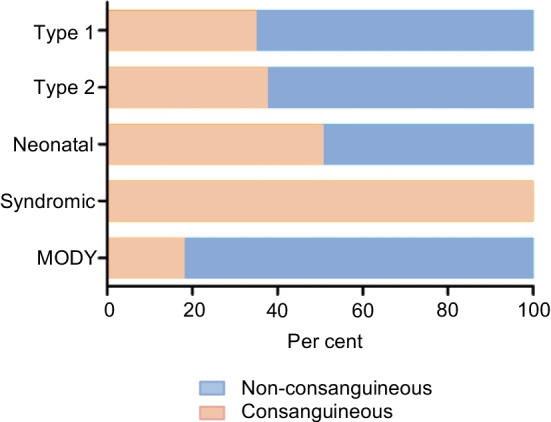
Distribution of consanguinity within each diabetes subtype

Of the 754 participants followed at the study centre, 714 (94.7%) were clinically classified as having type 1 diabetes. In total, 35% of these participants were from consanguineous families (Fig. 1). The median age of diabetes onset in type 1 diabetes was 7.7 years (IQR 4.2–10.2). All participants were treated using multiple daily insulin injections, and the median total daily insulin dose was 0.9 U/kg (IQR 0.73–1.3). The median BMI in this group was 16.6 kg/m^2^ (IQR 14.4–20.5) at the age of the most recent check-up. BMI SD normative values are not available for this population; therefore, WHO normative values were used when applicable [22].

Eight children (1.1%) had clinically assigned type 2 diabetes, three of whom (38%) were born to consanguineous parents (Fig. 1). The median BMI in this group was 20.1 kg/m^2^ (IQR 19.3–26.0) and median BMI SD was +1.8 SD (IQR +1.5 to +2.3) at the age of the most recent check-up (median 10.2 years, IQR 7.7–11.3). All participants in this group had acanthosis nigricans as a clinical sign of insulin resistance and were being treated with metformin, with three being on a combination of metformin and insulin.

Eleven participants (1.5%) had clinically defined MODY, two of whom (18%) were born to consanguineous parents (Fig. 1).

### Neonatal diabetes

A total of 14 participants (1.9%) had neonatal diabetes (50% consanguineous), with a mean age at diagnosis of 25 days (IQR 12.5–45) (Fig. 1).

Twelve of these participants were available for testing by WES, of whom ten had permanent neonatal diabetes. A pathogenic variant was found in 83% (10/12) of participants. In an additional proband, we found two novel compound heterozygous variants in the enhancer region of *PTF1A*. Even though one variant did not fulfil criteria for pathogenicity according to ACMG guidelines, it is possible that these variants may be the cause of diabetes in this participant.

The clinical phenotypes of these participants are shown in Table 1. Seven consanguineous participants (including one sibling pair) had homozygous variants in the *GLIS3*, *ZNF808* or *PTF1A* genes. Two participants from non-consanguineous families had a heterozygous pathogenic variant in *ABCC8* (transient diabetes) or *INS* (de novo) and one had a homozygous variant in the *PTF1A* enhancer region (Table 1).

**Table 1:** Relevant variants found in participants with neonatal and syndromic diabetes who underwent testing and participants’ clinical characteristics

Participant ID	Type of diabetes	Age at diagnosis	Reported consanguinity	Clinical characteristics in addition to diabetes	Gene (reference sequence in hg19)	Variant at cDNA level		Variant at protein level	Zygosity	ACMG	ROH percentage via WES	Reference if published
11298	Neonatal (permanent)	11 days	Yes	Congenital hypothyroidism, facial features (flat nasal bridge, long philtrum, low-set ears)	*GLIS3* (NM_001042413)	chr9:3785092_3828441del			HOM	P	6.6	Novel
11299	Neonatal (permanent)	2 months	Yes	Congenital hypothyroidism, facial features (flat nasal bridge, long philtrum, low-set ears)	*GLIS3*	chr9:3785092_3828441del			HOM	P	7.5	Novel
11682	Neonatal (permanent)	5 months	Yes	Prolonged jaundice after birth	*ZNF808* (NM_001039886)	c.1805_1806del		p.Lys602Serfs*9	HOM	P	12.5	Novel
13186	Neonatal (transient)	2 months	No	–	*PTF1A* (NM_178161)	chr10:23508314T>C			Comp. HET	VUS	0.0	Novel
				–	*PTF1A*	chr10:23508363A>C				LP		Novel
13192	Neonatal (permanent)	50 days	No	–	*INS* (NM_000207)	c.94G>A		p.Gly32Ser	HET	P	0.8	[28]
14020	Neonatal (permanent)	10 days	No	–	*PTF1A*	chr10:23508437A>G			HOM	P	2.0	[29]
14105	Neonatal (transient)	13 days	No	–	*ABCC8 (NM_000352)*	c.3592C>T		p.Pro1198Ser	HET	LP	0.3	Novel
14109	Neonatal (permanent)	6 months	Yes	–	*PTF1A*	c.571C>A		p.Pro191Thr	HOM	P	7.0	[23]
14341	Neonatal (permanent)	12 days	Yes	Prolonged jaundice after birth, abdominal distension	*PTF1A*	c.571C>A		p.Pro191Thr	HOM	P	8.5	[23]
14346	Neonatal (permanent)	35 days	Yes	Abdominal distension, hypogammoglobulinaemia, anaemia	*PTF1A*	chr10:23508437A>G			HOM	P	3.8	[29]
14348	Neonatal (permanent)	18 days	Yes	–	*PTF1A*	chr10:23508437A>G			HOM	P	5.9	[29]
10479	Syndromic	12 years	Yes	Coarse facial features, hypertrichosis, hyperpigmentation, acanthosis nigricans, overcrowded teeth, short stature, hypogonadotropic hypogonadism, severe insulin resistance	*INSR* (NM_00020)	c.2810C>T	p.Thr937 Met		HOM	P	3.5	[30]
11231	Syndromic	7 years	Yes	Short stature, suspected diabetes insipidus	*WFS1* (NM_006005)	c.2589C>G	p.Ile863 Met		HOM	LP	13.5	Novel
11232	Syndromic	12 years	Yes	Short stature, mild optic atrophy	*WFS1*	c.2589C>G	p.Ile863 Met		HOM	LP	13.0	Novel
11279	Syndromic	8 years	Yes	Short stature, camptodactyly, hepatosplenomegaly, aortic regurgitation, myopia, lacrimal duct obstruction	*SLC29A3 (*NM_018344)	c.1041delC	p.Leu349Serfs*56		HOM	P	9.9	[31]
14117	Syndromic/type 2 diabetes	11 years	Yes	Joint stiffness, genu valgum, claw-hand deformity, mild intellectual disability	*GNPTG* (NM_032520)	c.494dupC	p.T165fs		HOM	P	3.5	[32]

Comp. HET, compound heterozygous variant; HOM, homozygous variant; HET, heterozygous variant; LP, likely pathogenic; P, pathogenic; ROH, runs of homozygosity/per cent of the autosome covered by homozygous regions at least 3 Mbp in size; VUS, variant of uncertain significance

The sibling pair (11298 and 11299 in Table 1) had a large homozygous deletion in the *GLIS3* gene causing neonatal diabetes and congenital hypothyroidism. On examination, these participants had distinct facial features typical for this genetic variant (flat nasal bridge, long philtrum, low-set ears, eye protuberance). Growth parameters were normal.

The bi-allelic variant in *ZNF808* was found in a participant born preterm (32nd gestational week). He was diagnosed with neonatal diabetes at age 5 months (adjusted age). He had prolonged jaundice with conjugated hyperbilirubinaemia until the fourth month of life, with failure to thrive, and at 5 months of age he was hospitalised for diabetic ketoacidosis.

We observed a high percentage of pathogenic *PTF1A* variants among probands with genetically confirmed neonatal diabetes (50% of all pathogenic variants), including variants in the enhancer region. Two non-related participants from consanguineous families and one non-consanguineous participant were homozygous for the chr10:23508437A>G variant in the enhancer region (Table 1). Another participant with transient neonatal diabetes was compound heterozygous for two variants in the *PTF1A* enhancer region. One variant (chr10:23508363A>C) was classified as likely to be pathogenic while the other (chr10:23508314T>C) was classified as being a variant of uncertain significance (VUS); both have not been published previously. All participants who were positive for pathogenic *PTF1A* enhancer variants were diagnosed earlier than other children with neonatal diabetes, at 12–35 days of age.

Two non-related participants from consanguineous families had a homozygous hypomorphic coding variant in *PTF1A* (p.Pro191Thr). This variant has been published as causing pancreatic aplasia/hypoplasia with reduced exocrine function and normal neurological function [23]. One of these participants was diagnosed with diabetes at 12 days of age and the other was diagnosed at 6 months of age.

### Syndromic diabetes

**Fig. 2:**
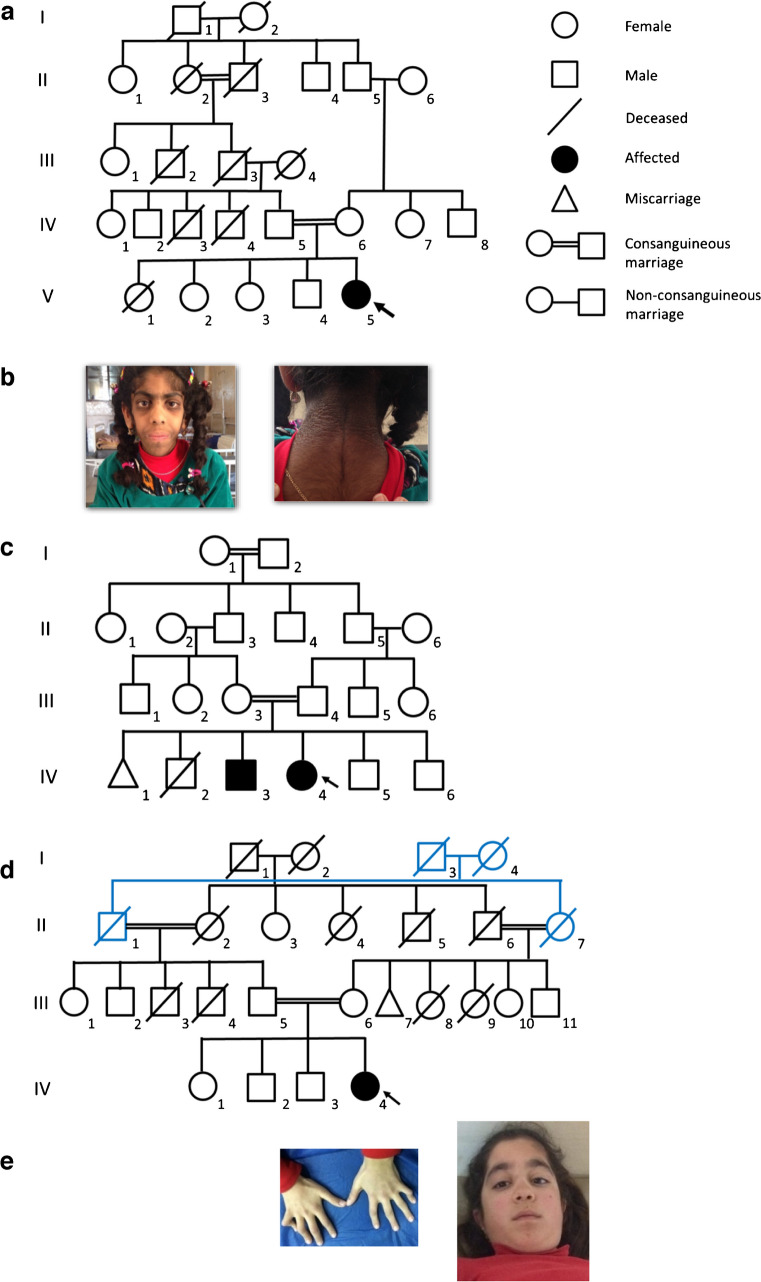
Participant pedigrees and images of participants with syndromic diabetes. Full facial images are shown with parental permission. (**a**) Pedigree of the participant with the *INSR* variant (V-5); (**b**) dysmorphic facial features in V-5 from (**a**), including hypertrichosis and hyperpigmentation; (**c**) pedigree of the siblings with the *WFS1* variant (IV-3 and IV-4); (**d**) pedigree of the participant with the *SLC29A3* variant (IV-4); II-1 and II-7 are siblings and II-2 and II-6 are siblings; (**e**) mild facial dysmorphy and camptodactyly in IV-4 from (**d**)

Seven participants (0.9%) had syndromic diabetes, all of whom were from consanguineous families (Fig. 1). A (likely) pathogenic variant was found in four of seven (57%) participants (Table 1). All pathogenic variants were detected in the homozygous state (in *INSR*, *SLC29A3* and *WFS1* [in a sibling pair]). All participants had relatively distinct phenotypic features (Table 1, Fig. 2). The most apparent features were present in the participant with the *INSR* pathogenic variant, who had coarse facial features, severe hypertrichosis, acanthosis nigricans, a height of −3.2 SD at 13 years of age and a bone age delay of 4 years.

Pathogenic variants in *SLC29A3* cause histiocytosis–lymphadenopathy plus syndrome or H-syndrome. The participant with this pathogenic variant had a short stature at 12 years of age (−3.4 SD), autoantibody-negative diabetes, hepatosplenomegaly, camptodactyly and mild aortic regurgitation. She also had a sensorineural hearing impairment and myopia.

Two siblings who were diagnosed with diabetes at 7 and 12 years of age and who had a short stature had a homozygous mutation in *WFS1* causing Wolfram syndrome. The older sibling had partial optic atrophy with normal vision and the other had polyuria due to partial diabetes insipidus.

## Discussion

Our retrospective single-centre study of 754 children with diabetes in a highly consanguineous population found that 95% had a clinical diagnosis of type 1 diabetes. To our knowledge, this is the first such study from a region with a high consanguinity rate of up to 44%. The other subtypes of diabetes were much less common: 1.9% had neonatal diabetes, 1.5% had clinically defined MODY, 1.1% had type 2 diabetes and 0.9% had syndromic diabetes. In a study from Turkey (20% consanguinity), 84% of participants were classified as having type 1 diabetes and 5.7% as having type 2 diabetes [8]. A more recent Turkish study focused on children with monogenic diabetes and found a prevalence of 3.1%, which was similar to that in a cohort from the UK (lower rate of consanguinity) [20]. Our study found the prevalence of monogenic diabetes to be 4.2% among all participants followed at the single centre. However, because of the lack of standard antibody testing in those with diabetes, a proportion of individuals with MODY subtypes (such as *HNF1A*/*HNF4A*-MODY) may be misdiagnosed as having type 1 diabetes. Therefore, the actual percentage of individuals with MODY in this population may be higher, which could lead to a greater overall prevalence of monogenic diabetes. The higher prevalence may be attributed to the presence of more recessive forms of diabetes due to the high percentage of consanguinity.

The rate of consanguinity in the study region of Sulaimani has not been formally documented, but a study investigating congenital heart disease in the same region reported 41% consanguinity among the study population [12]. In a 2010 study evaluating consanguinity in the region of Baghdad, Iraq, the rate was 44% [13]. There was a significant association between consanguinity and sociodemographic characteristics, such as differences in rates among urban and rural populations. Furthermore, in a study from north-west Iran, which is geographically close to Kurdistan, Iraq, the rate of consanguinity was 39.1% [14]. These data can be used to make an approximation of the possible rate of consanguinity in the region of Sulaimani.

The consanguinity rate among participants followed at our centre was 36.5%. This is close to the rate among the majority group of participants—those with type 1 diabetes (35%). Although previous studies have shown that the prevalence of type 1 diabetes is not influenced by consanguinity, there is a higher risk of development of type 1 diabetes if there is a history of diabetes in first cousin parents [24]. We confirmed that there was no statistically significant association between consanguinity and type 1 diabetes.

It would be expected that a child from a first cousin marriage would have 6% of the genome covered in ROH. Probands with positive consanguinity who underwent genetic testing (all from reported first cousin marriages) had 3.8–13.5% ROH (Table 1). The participants with no reported consanguinity had 0.0–2.0% ROH. However, because only coding regions were analysed by WES, there may be additional ROH in non-coding regions of the genome.

A genetic diagnosis was identified in 83% of participants with neonatal diabetes who were available for testing by WES (i.e. 10/12). Among these, 80% were homozygous for pathogenic variants causing the disease. There were only two participants with transient neonatal diabetes, with variants in *ABCC8* and *PTF1A* (VUS). In a study comparing genetic causes of neonatal diabetes among consanguineous and non-consanguineous populations, the most common cause of neonatal diabetes among participants born to consanguineous parents was recessive *EIF2AK3* gene variations causing Wolcott–Rallison syndrome, whereas in non-consanguineous populations pathogenic variants in the *KCNJ11* and *ABCC8* genes accounted for the majority of cases (46%). These genes (*KCNJ11* and *ABCC8*) accounted for only 12% of cases in the consanguineous group [10]. Moreover, there was a much higher incidence of recessive forms of neonatal diabetes in consanguineous regions, which we also found in our study.

The overall spectrum of monogenic diabetes genes found in our study population was different from what is found in non-consanguineous populations [10, 25]. We observed homozygous, causal variants in genes such as *PTF1A*, *GLIS3*, *INSR* and *SLC29A3*, which are uncommon in non-consanguineous populations. Consanguineous populations may differ in their genetic burden because of founder effects and the frequency of heterozygotes in potentially pathogenic genes. This is apparent in regard to the number of *PTF1A* variants in our study population. In comparison, the study from Turkey had a predominance of recessive variants in the *WFS1* and *SLC19A3* genes [20]. Therefore, it can be concluded that each consanguineous population is unique, which can allow specific insights into the genetics of conditions such as monogenic diabetes [26].

Most participants with syndromic diabetes in our cohort had relatively distinct phenotypic features suggestive of a monogenic condition, for example the participant with the *INSR* pathogenic variant who had features typical of Rabson–Mendenhall syndrome. At 63 days, she underwent a bilateral oophorectomy owing to the presence of bilateral cysts and the suspicion of a juvenile granulosa cell tumour. This led to hypergonadotropic hypogonadism with absent pubertal development. In our participant, surgery was carried out very early; however, individuals with *INSR* defects can present peripubertally with features resembling polycystic ovary syndrome or adrenache and, if genetic testing is carried out in a timely manner, invasive measures can be avoided.

In the participant with the *SLC29A3* variant causing H-syndrome, the genetic diagnosis was crucial for correct management. Initial evaluation of this participant was performed because of their short stature, hepatosplenomegaly and camptodactyly, which led to examination for mucopolysaccharidosis, revealing mildly decreased levels of alpha-iduronidase. The diagnosis of mucopolysaccharidosis type I was confirmed and the participant was on expensive enzyme therapy (with no effect) until genetic diagnosis.

One participant who was suspected of having syndromic diabetes had a pathogenic variant in the *GNPTG* gene, confirming a diagnosis of mucolipidosis gamma. With regard to his diabetes, we did not find a causal variant. He is currently being treated with metformin, so it is uncertain if his diabetes could be clinically classified as type 2 diabetes or if there is an impact from an unrecognised gene variant.

This raises an interesting point about the diagnosis and classification of individuals with syndromic diabetes, especially in consanguineous regions. It can be argued that the usual set criteria for syndromic diabetes can be misleading in some cases [20]. In addition, in consanguineous families, individuals may have a single gene condition causing the extra-pancreatic phenotype and concurrently develop type 1/type 2 diabetes. Another possibility is the presence of multiple causative homozygous variants causing two conditions, including monogenic diabetes.

Our results showed that consanguinity was significantly associated with syndromic diabetes (*p*=0.0023) but not with other diabetes subtypes. Therefore, genetic testing in individuals with a suspicion of syndromic diabetes from consanguineous regions is crucial. However, setting criteria for genetic testing in such individuals is restricted by factors such as limited antibody testing. Our testing criteria (see Methods) yielded a high percentage of positive results. We observed that the presence of short stature and hepatosplenomegaly were crucial in finding monogenic diabetes variants in certain participants. A recent study also found that the presence of specific non-autoimmune extra-pancreatic features (deafness, anaemia and developmental delay) markedly improved the identification of autosomal recessive monogenic diabetes [20]. Consanguinity of parents was a helpful identifying factor as well [20]. Taken together, we suggest testing all individuals matching our selection criteria, with special emphasis on the presence of short stature, hepatosplenomegaly, deafness, anaemia and/or developmental delay.

Enabling genetic testing of people with diabetes in consanguineous populations is important in improving diagnostic criteria for monogenic diabetes [20]. In addition, studies in consanguineous populations have led to the ongoing discovery of novel genes and pathophysiological pathways [26]. One pathogenic variant among our cohort was in the *ZNF808* gene (Table 1), which was identified very recently as causing neonatal diabetes in consanguineous families [27].

Our study provides a new rare insight into the influence of consanguinity on diabetes subtypes in Kurdistan, Iraq, and on the spectrum of genes that are causative of monogenic diabetes (specifically neonatal and syndromic diabetes). The main limitations of our research are the lack of a computerised system for collecting and maintaining patient data, leading to possible transcription errors and missing data for some participants. Furthermore, a lack of records on family history of diabetes and a lack of or limited access to certain laboratory tests such as routine antibody testing and evaluation of C-peptide levels were limiting factors in calculating genetic risk scores. Subtypes were mostly defined clinically by the attending physicians. Genetic testing in those with a clinical suspicion of MODY was not carried out because of a lack of informed consent and available blood samples for genetic testing in many. Furthermore, we believe that MODY prevalence was underestimated because of the reasons mentioned above, the lack of a comprehensive family history of diabetes and the lack of preventive check-ups to identify cases of hyperglycaemia.

### Conclusions

Our single-centre study provides a unique insight into the prevalence and genetic causes of neonatal and syndromic diabetes in a highly consanguineous population. Our data confirm that, even in such populations, type 1 diabetes is the prevailing paediatric diabetes subtype. It was found that syndromic diabetes is strongly associated with consanguinity. The causative gene in monogenic diabetes was successfully elucidated in 83% of participants with neonatal diabetes and 57% of participants with syndromic diabetes. Homozygous variants made up 80% of all pathogenic variants identified. The spectrum of causative genes (*PTF1A*, *GLIS3*, *WFS1*, *INSR*, *SLC29A3*, *ZNF808*, *ABCC8*, *INS*) is markedly different from the monogenic diabetes genes seen in non-consanguineous cohorts, and also different from those seen in other consanguineous populations. In addition, we observed that phenotypic features such as short stature and hepatosplenomegaly may be important diagnostic criteria for syndromic diabetes in consanguineous populations, in whom diagnosis can be complicated due to the presence of concomitant conditions.

## Data Availability

The data that support the findings of this study are not openly available because of institutional ethics restrictions but are available from the corresponding author on reasonable request.

## Abbreviations

ACMG: American College of Medical Genetics and Genomics
NGS: Next-generation sequencing
ROH: Runs of homozygosity
VUS: Variant of uncertain significance
WES: Whole-exome sequencing
